# Evidence-Based Novel Changes in Prevalence and Symptom Characteristics of Spleen Deficiency Syndrome in Persons of Varied Health Status and Different Ages: A Cross-Sectional Observational Study

**DOI:** 10.1155/2014/818235

**Published:** 2014-11-11

**Authors:** Yin Zhang, Yue Liu, Xian-ping Li, Jian Li, Xiao-feng Li, Liang Chen, Yu-yong Jiang, Hao Yu, Ning Shi, Mei Han, Hui Ye, Zi-han Lin, Yue-zhou Chen, Fu-sheng Liu, Xia Ding

**Affiliations:** ^1^First Clinic College of Medicine, Beijing University of Chinese Medicine, Dongcheng District, Beijing 100700, China; ^2^Second Clinic College of Medicine, Beijing University of Chinese Medicine, Fengtai District, Beijing 100078, China; ^3^Department of Development Planning, Beijing University of Chinese Medicine, Chaoyang District, Beijing 100029, China; ^4^School of Basic Medical Science, Beijing University of Chinese Medicine, Chaoyang District, Beijing 100029, China; ^5^Department of Internal Medicine, Changping District Hospital of Integrative Medicine, Changping District, Beijing 102208, China; ^6^Department of Traditional Chinese Medicine, Ditan Hospital Affiliated to Beijing Capital Medical University, Chaoyang District, Beijing 100015, China; ^7^Department of Health Administration, Electric Power Teaching Hospital of Capital Medical University, Fengtai District, Beijing 100073, China; ^8^Center for Evidence Based Medicine, Beijing University of Chinese Medicine, Chaoyang District, Beijing 100029, China; ^9^Department of Acupuncture, Quanzhou Hospital of Traditional Chinese Medicine, Licheng District, Quanzhou 362000, China; ^10^China Institute for History of Medicine and Medical Literature, China Academy of Chinese Medical Sciences, Beijing 100700, China; ^11^Department of Medical Administration, Beijing University of Chinese Medicine, No. 11, North Third Ring Road, Chaoyang District, Beijing 100029, China

## Abstract

Deficiency of the organs is a vital pathophysiologic characteristic in the elderly. A core TCM aging theory is known as aging caused by spleen deficiency syndrome (SDS) that can be found in ancient and modern literature. The key objectives of this study were to establish a full-scale trial to evaluate the prevalence, symptom severity, frequency, and distribution of SDS in different age groups as related to health status (healthy, subhealthy, and chronic disease) to elucidate the role of spleen deficiency in the aging process and deterioration of health status. This cross-sectional observational study was conducted in 4 hospitals in China. 1390 participants aged 20–79 were interviewed by investigators who completed questionnaires recording prevalence, severity, and frequency of symptoms as well as other relevant information. The results revealed that prevalence and symptom characteristics of SDS showed regularities with increasing age and deteriorating health status. It supports the TCM concept that spleen deficiency is an important mechanism of aging, subhealth, and chronic diseases. Early recognition of the warning signs and symptoms of SDS may lead to intervention and even prevention strategies for subhealth and chronic diseases as well as promotion of healthy aging.

## 1. Introduction

Aging is a spontaneous, long-term, gradual, complex, and inevitable process recognized by modern medicine as physiologic deterioration and decline. The key tenet of traditional Chinese medicine (TCM) is the Chinese philosophic concept of holism [1], with aging an ongoing pathophysiologic process that occurs in response to influences external to the organism as well as to internal functional decline of the organs, meridians, flow of* qi* and blood, and* yin* and* yang.*

Deficiency of the organs is a vital pathophysiologic characteristic in the elderly. A core TCM aging theory is known as aging caused by spleen deficiency. Evidence supporting this theory can be found in ancient and modern literature [2–7]. Earliest mention may have been in the* Yellow Emperor's Inner Classic *(*Huangdi Nei Jing*). Since ancient times dating to the Han dynasty (200s CE), this theory has played a crucial role in preventing disease, promoting longevity, and the delivery of daily health care in China. Modern clinical and laboratory studies have explored the essence of spleen deficiency syndrome (SDS) as well as the correlation between SDS and the digestive system [8, 9].

Chinese society is aging at an unprecedented rate [10, 11]. There will be an estimated 438 million elderly in China by 2050, occupying 21.8% of the world's elderly population (2 billion) [12, 13]. Although longevity is a symbol of good health and shows global health improvement, the growing demand for health services for the aging population is a major challenge for medical and social services not only in China, but also worldwide [13].

The issue of health, subhealth (suboptimal health), chronic disease, and geriatrics has attracted widespread attention in China [14–18] and has been clearly defined by medical professionals [19–21]. In terms of TCM, health is defined as optimal physiologic functioning in self-regulation, adaptation to the environment, resistance against pathogens, and self-recovery from illness. Subhealth and chronic disease are the result of physiologic dysfunction that can be differentiated into TCM pathogenic patterns, or syndromes, of excess or deficiency of the organs,* qi*, blood, and* yin* and* yang*.

Epidemiologic studies have demonstrated the correlation between SDS and health status (subhealth and chronic disease). Results show the percentage of the spleen deficiency population quadruples for both males and females between 20–30 years old and 50–60 years old, a growth rate of more than 10 percent per decade demonstrating that compared with the young and middle-aged the elderly have a much higher prevalence of SDS [14, 15, 22–24]. SDS is one of the most common syndromes in the subhealthy and chronic disease populations and is complicated by other complex syndromes [25–28].

There are several shortcomings in existing epidemiologic studies on SDS though progresses have been made in gaining a better understanding of this syndrome as related to aging. None of these studies describe age-related changes of SDS in symptom severity, frequency, and distribution in addition to prevalence. None of the studies compare the differences in varied health status populations. In addition, their poor quality control in statistical analyses and study design precludes reliability of their results.

In this context, we designed the present study to evaluate the prevalence, symptom severity, frequency, and distribution of SDS in different age and health status (healthy, subhealthy, and chronic disease) groups so as to address these limitations. Our key objective was to elucidate the role of spleen deficiency in the aging process and deterioration of health status objectively and systematically.

## 2. Methods

### 2.1. Study Design and Participants

This cross-sectional study was conducted between April 2009 and August 2013. Inpatients, outpatients, and individuals were recruited by simple random sampling from hospitals in Beijing and Xiamen, China: Dongzhimen Hospital Affiliated to Beijing University of Chinese Medicine; Xiamen Traditional Chinese Medicine Hospital; Peking Union Medical College Hospital Affiliated to the Chinese Academy of Medical Sciences; and Beijing Hospital Affiliated to the National Health and Family Planning Commission of China. All participants involved in this survey signed consent forms, the numbers of which were evenly distributed in all four seasons.

### 2.2. Diagnostic Criteria

Diagnostic criteria of SDS were based on the China Association of Integrative Medicine Reference Guideline for Traditional Chinese Medicine Deficiency Syndrome Differentiation [29]. For this study, participants presenting with at least three of the five following signs and symptoms, loose stools, abdominal distension following meals, sallow complexion, loss of appetite, weight loss, and general weakness, were diagnosed with SDS.

Diagnostic criteria of subhealth were based on* Clinical Guidelines of Chinese Medicine on Subhealth* published by the China Association of Chinese Medicine [20]. After physical examination and appropriate diagnostic testing, patients were diagnosed as subhealthy when they presented with several defined somatic, psychologic, or social adaptation symptoms that had lasted at least 3 months.

Diagnostic criteria of chronic disease were specialty-based. Diagnoses were reached after detailed evaluation, including medical history, physical examination, and diagnostic testing.

Participants who meet the criteria of neither subhealth nor chronic disease were diagnosed as healthy.

### 2.3. Inclusion and Exclusion Criteria

Inclusion criteria were 20–79 years of age; signed informed consent; meeting diagnostic criteria of health, subhealth, or chronic disease; willingness to respond to investigator queries truthfully while completing the clinical observation questionnaires.

Potential participants who did not meet the age requirement or any of the above-mentioned diagnostic criteria, were unwilling to sign the consent form, were unwilling or unable to complete questionnaires, or had mental disorders were excluded at screening.

### 2.4. Questionnaire Content and Administration

A clinical observation questionnaire was designed for survey of participants to capture data on TCM symptoms based on deficiency syndrome differentiation guidelines [29]. In addition, demographic information (name, gender, and age), disease information (chief complaint, present and past medical history), and subhealth-related information (physical, psychologic, and social adaptation symptoms) were also included in this questionnaire. A list of detailed definitions of severity and frequency of symptoms was prepared for investigators and participants to refer to as additional file to questionnaires. To ensure quality control, investigators at each medical center received training on standard operating procedure before the study began. Each participant was interviewed by two or more resident TCM physicians based on questionnaire after enrollment in the study with at least two senior physicians supervising each interview session.

### 2.5. Statistical Analysis

Comparative analysis of SDS prevalence in different age and health status groups was expressed as composition ratio and performed by frequency analysis and chi-square test. Comparative analysis of quantitative scores of the severity and frequency of symptoms was performed by rank-sum test. A probability of *P* < 0.05 was considered statistically significant. Regression analysis of correlation of SDS occurrence and potential factors was performed by nonconditional binary logistic stepwise regression of numerical variables. Significance level of introducing and removing variables was 0.05 and 0.10, respectively. All statistical analyses were performed by SPSS software (*version 17.0, SPSS Inc., Chicago, IL*) in this study.

## 3. Results

### 3.1. Characteristics of Participants

1495 questionnaires were distributed and a total of 1427 (95.45%) were completed. 1390 forms were deemed eligible for the study after eliminating questionnaires with incomplete information, with a rate of 97.4%. In terms of age distribution, 508 participants were aged 20 to 39, 472 were aged 40 to 59, and 410 were aged 60–79. As for health status distributions, 366 were characterized as healthy, 745 were subhealthy, and 279 were experiencing chronic disease. There were 682 males and 708 females, a gender ratio of 0.963.

### 3.2. Prevalence of SDS in the Same Health Status but Different Ages

**Figure 1:**
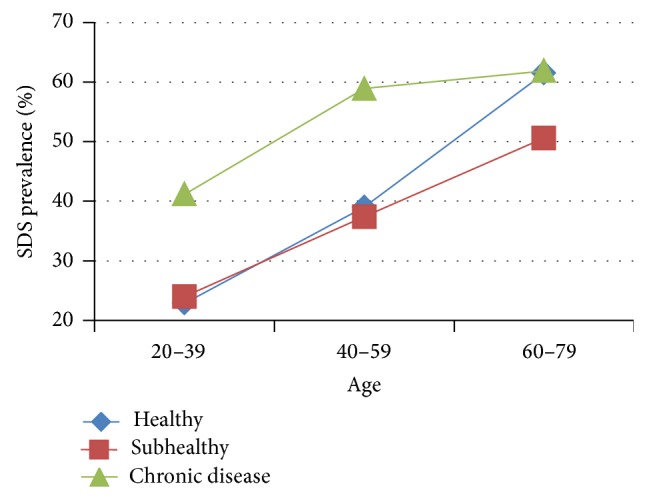
Trend in SDS prevalence with increasing age. Data from 3 independent age groups were presented as percentage.

Prevalence of SDS in the same health status group showed a significant rising trend with increasing age (Table 1, Figure 1). Prevalence in persons 40–59 years of age was higher than in persons 20–39 years of age and was lower than in persons 60–79 years of age (*P* = 0.012 and *P* = 0.009, resp.). In the subhealthy group, SDS prevalence demonstrated a similar rising tendency as age increased. Comparisons between the 20–39 and 40–59 age groups and between the 40–59 and 60–79 age groups were both statistically significant (*P* = 0.001 and *P* = 0.018, resp.). SDS prevalence in persons with chronic disease did not appear significantly different between the 40–59 and 60–79 age groups (*P* > 0.05). However, compared with these two groups, prevalence in the 20–39 age group was lower (*P* = 0.034 and *P* = 0.011, resp.).

### 3.3. Prevalence of SDS in Varied Health Status within the Same Age Group

**Figure 2:**
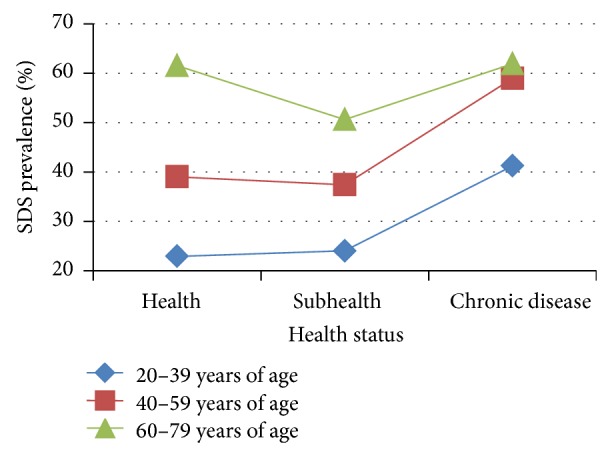
Trend in SDS prevalence with deterioration of health. Data from 3 independent health status groups were presented as percentage.

In each age group, SDS prevalence varied by health status (Table 2, Figure 2). In the 20–39-year and 40–59-year age groups, compared with the healthy and subhealthy groups, SDS prevalence rose when physical condition worsened, while in the 60–79-year age group there was no such trend. In the 20–39-year age group, persons with chronic disease had a higher prevalence of SDS compared with healthy and subhealth individuals (*P* = 0.005 and *P* = 0.011, resp.). In the 40–59-year age group, SDS prevalence of persons with chronic disease was also higher than in healthy and subhealthy persons (*P* = 0.013 and *P* = 0.003, resp.). In the 60–79-year age group, prevalence was even lower in subhealthy participants compared with the healthy and chronic disease groups (*P* < 0.001 and *P* = 0.003, resp.). In the 20–39 and 40–59-year age groups, comparisons of SDS prevalence between healthy and subhealthy participants were not significantly different (*P* > 0.05). However, in the 60–79-year age group, comparison of SDS prevalence between healthy participants and those with chronic disease also showed no significant difference.

### 3.4. Severity and Frequency of SDS-Related Symptoms

Definitions of severity and frequency of the five spleen deficiency symptoms (loose stools, abdominal distension following meals, sallow complexion, loss of appetite, weight loss, and general weakness) were attached to the questionnaire for participants and investigators to refer to. Symptom severity was assigned the following scores: asymptomatic = 0; mild = 1; moderate = 2; severe = 3. Symptom frequency was assigned the following scores: never = 0; occasional = 1; intermittent = 2; often = 3.

**Figure 3:**
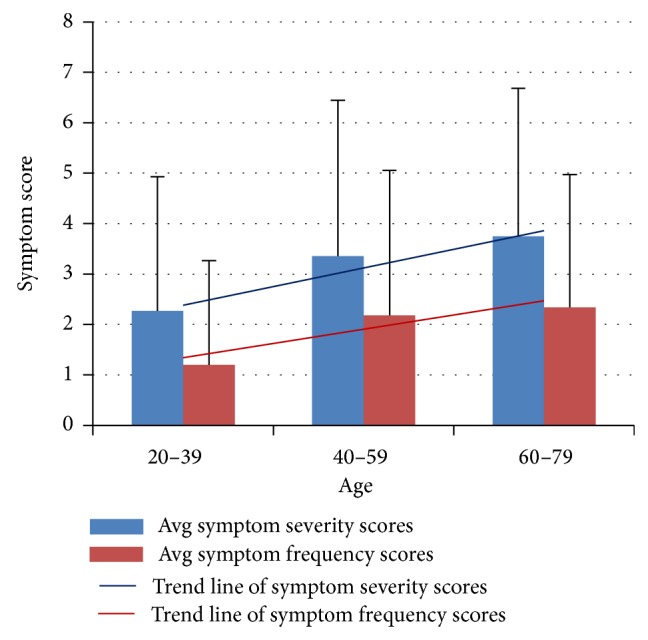
Trends in severity and frequency of SDS-related symptoms with increasing age. Data from 3 independent age groups were presented as mean (standard deviation).

*SDS Symptom Severity and Frequency at Different Ages.* Symptom severity scores in the 40–59 age group were higher than in the younger age group (*P* < 0.001). Symptoms were more severe in the 60–79 age group than in the middle-aged (*P* < 0.001) (Table 3, Figure 3). Comparisons of symptom frequency scores among the three groups were all statistically significant (*P* < 0.001), indicating that SDS-related symptoms present more frequently with increasing age (Tables 3 and 4, Figure 3).

**Figure 4:**
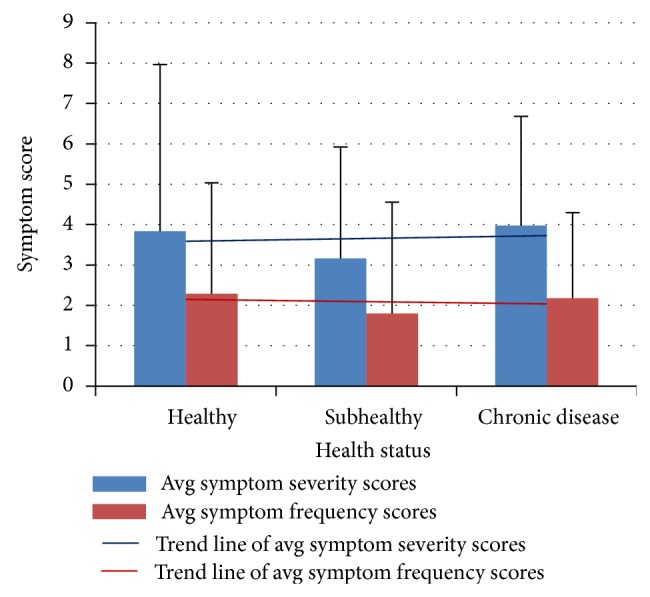
Trends in severity and frequency of SDS symptoms with deteriorating health. Data from 3 independent health status groups were presented as mean (standard deviation).

*SDS Symptom Severity and Frequency as Related to Health Status.* Symptom severity and frequency scores were not significantly different between the three health status groups (*P* > 0.05) (Tables 5 and 6, Figure 4).

### 3.5. Prevalence of SDS and Potential Factors

Unconditional binary logistic stepwise regression of numerical variables was applied to evaluate the correlation of SDS prevalence and other potential related factors including age, health status, and symptoms. Significance level of entered and removed variables was 0.05 and 0.10, respectively.

Independent variables were *χ*_1_ gender, *χ*_2_ age, *χ*_3_ health status, *χ*_4_ loose stools, *χ*_5_ abdominal distension after meal, *χ*_6_ sallow complexion, *χ*_7_ loss of appetite, and *χ*_8_ weight loss and weakness. Dependent variable *y* represented whether or not participants met SDS diagnostic criteria. The logistic regression equation was

(1) y=5.903−0.216χ2−0.512χ3−1.430χ4 −1.176χ5−1.482χ6−1.679χ7−0.405χ8.

Results showed that age, health status, loose stools, abdominal distension after meal, sallow complexion, loss of appetite, and weight loss and weakness contributed to the prevalence of SDS (*P* < 0.05); none of these variables had an OR > 1. Gender did not affect prevalence of SDS (*P* > 0.05).

### 3.6. SDS Symptoms as Related to Health Status and Different Ages

**Figure 5:**
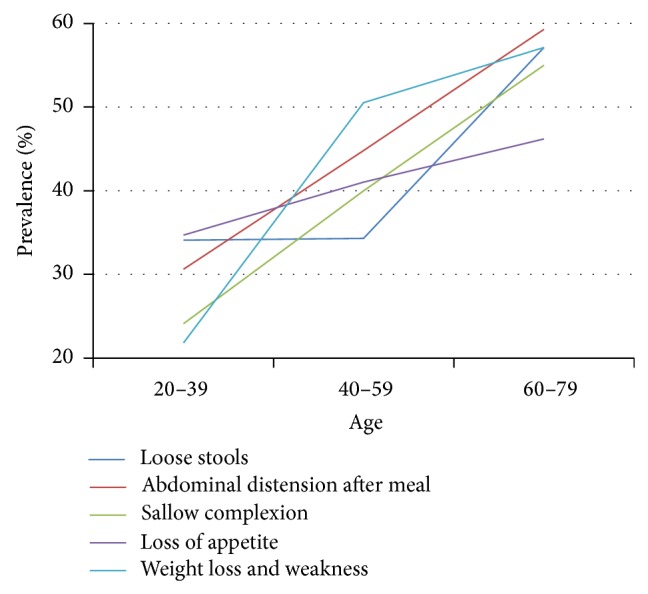
Distribution of SDS-related symptoms in healthy people of different ages. Data from 3 independent age groups were presented as percentage.

Among healthy individuals, prevalence of SDS symptoms was markedly higher with increasing age. Prevalence of loss of appetite increased less precipitously than other symptoms (Table 7, Figure 5). Prevalence of loose stools in persons 60–79 years of age was almost twice that of 20–30 and 40–59-year-olds. Prevalence between these two latter age groups remained steady.

**Figure 6:**
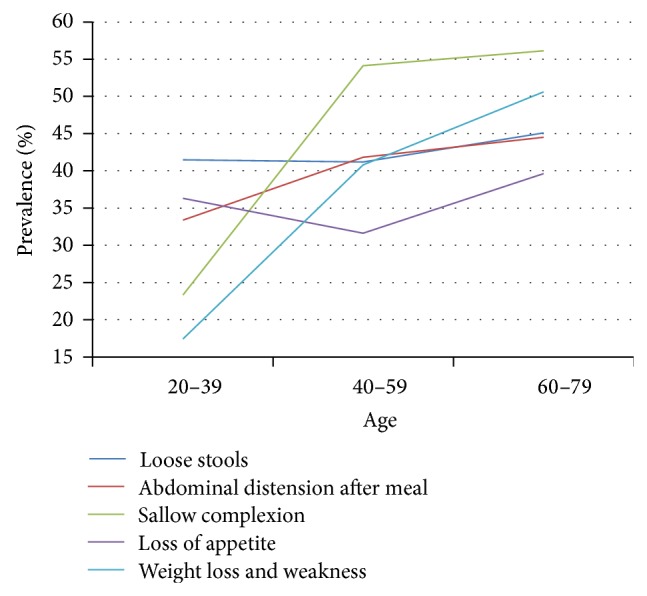
Distribution of SDS-related symptoms in subhealthy people of different ages. Data from 3 independent age groups were presented as percentage.

In the subhealthy group, prevalence of abdominal distension and loose stools rose slowly with increasing age. Sallow complexion and weight loss and weakness accelerated significantly between 20–39 and 40–59 years of age and slowed markedly between 40–59 and 60–79 years of age. Prevalence of loss of appetite declined between 20–39 and 40–59 years of age but increased between 40–59 and 60–79 years of age (Table 7, Figure 6).

**Figure 7:**
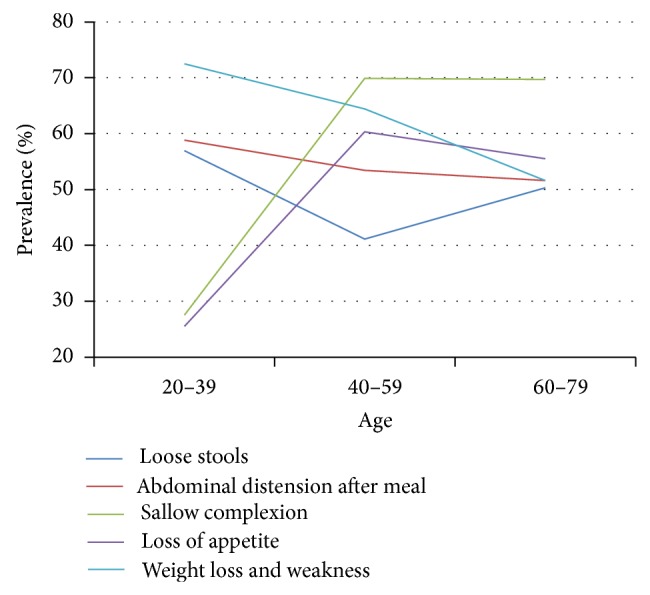
Distribution of SDS symptoms in persons with chronic disease at different ages. Data from 3 independent age groups were presented as percentage.

Among persons with chronic disease, prevalence of both abdominal distension and weight loss and weakness exhibited decline with increasing age. Loose stools also declined between the 20–39- and 40–59-year-olds but then increased between the 40–59- and 60–79-year-olds. Loss of appetite rose sharply between 20–39 and 40–59 years of age, but this symptom improved between ages 40–59 and 60–79 (Table 7, Figure 7).

**Figure 8:**
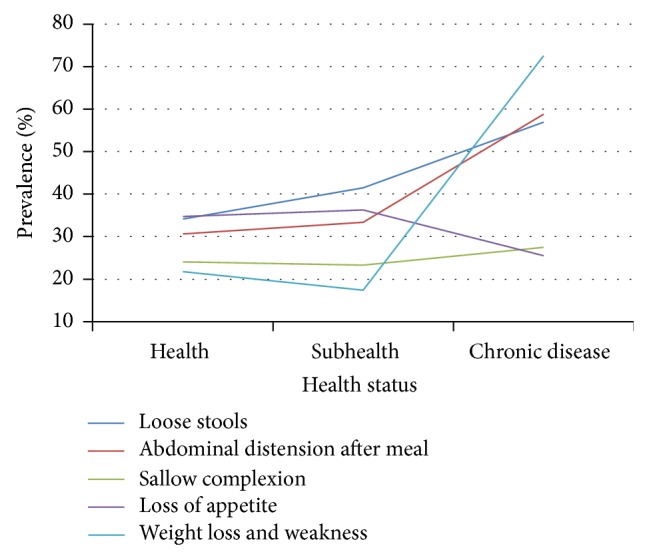
Distribution of SDS symptoms in 20–39 age group of different health status. Data from 3 independent health status groups were presented as percentage.

Prevalence of SDS symptoms exhibited certain trends as health status deteriorated. Among the 20–39 age group, prevalence of loose stools, abdominal distension, and sallow complexion rose slowly, with loss of appetite then decreasing and abdominal distension increasing markedly in the chronic disease group (Table 7, Figure 8). Prevalence of weight loss and weakness declined slowly between healthy and subhealthy persons and increased dramatically between subhealthy and chronic disease individuals.

**Figure 9:**
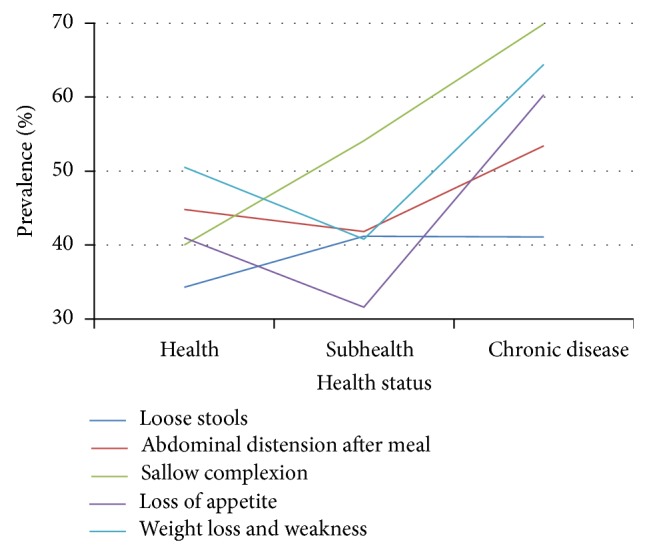
Distribution of SDS symptoms in 40–59 age group of different health status. Data from 3 independent health status groups were presented as percentage.

In the 40–59 age group, prevalence of weight loss and weakness, loss of appetite, and abdominal distension showed decline between healthy and subhealthy persons, followed by sharp increase in the chronic disease group. Prevalence of sallow complexion increased steadily as health status worsened (Table 7, Figure 9).

**Figure 10:**
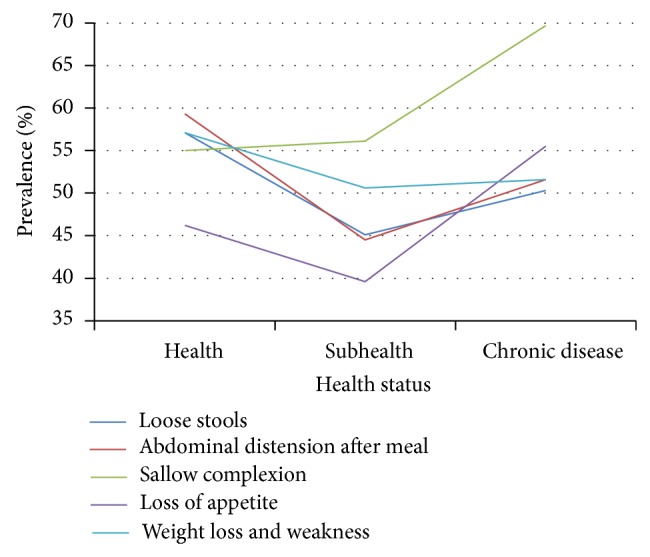
Distribution of SDS symptoms in 60–79 age group of different health status. Data from 3 independent health status groups were presented as percentage.

In persons 60–79 years old, prevalence of all symptoms except sallow complexion declined between the healthy and subhealthy groups. Prevalence of sallow complexion rose slightly between healthy and subhealthy individuals and then rose markedly in the chronic disease group (Table 7, Figure 10). In all three health status groups, sallow complexion had the highest prevalence of all SDS symptoms (Table 7, Figures 8–10).

## 4. Discussion

This study aimed to determine how the TCM syndrome and symptoms of spleen deficiency manifest during human aging and health status deterioration. Our results found that as healthy and subhealthy persons aged, SDS prevalence rose steadily. By the time these two groups reached seniorhood, they exhibited nearly the same SDS prevalence as seniors with chronic disease. In the subhealthy group, prevalence approached the level of seniors, and in the healthy group, prevalence nearly equaled that of seniors, thus showing that there is a close relationship between SDS and aging. In addition, spleen deficiency may play a crucial role in chronic disease pathogenesis since as compared with young and middle-aged healthy and subhealthy individuals persons with chronic disease of the same ages had a much higher prevalence of SDS.

Interestingly, SDS prevalence did not increase significantly between the middle-aged and seniors in persons with chronic disease. Middle age may be the turning point of the effects of SDS in persons with chronic disease, possibly indicating that spleen deficiency has less of an impact on health when they are older compared with healthy and subhealthy individuals of the same age.

In 20–39-year-olds, SDS prevalence increased slightly as they became subhealthy, followed by a dramatic increase when they developed chronic disease. In the 40–59- and 60–79-year-olds SDS prevalence remained stable between the healthy and subhealthy statuses, and the decrease was significant between the subhealthy and chronic disease statuses. Explanation for these phenomena may be that the etiology and pathogenesis of aging as health declines from a state of subhealth to chronic disease are more complicated than predicted in the middle-aged and elderly. Except for spleen deficiency as a directly related factor in aging, which is mentioned explicitly in TCM classics, other factors may play roles in these processes, especially in seniorhood.

SDS symptom severity scores showed a rising trend with increasing age and deteriorating health status, while such tendency did not exist for symptom frequency scores. It appears that age, health status, loose stools, abdominal distension after meal, sallow complexion, loss of appetite, weight loss, and weakness were contributing factors to SDS. Distribution of SDS symptoms in certain health status and age stages also showed dramatic irregularities.

With an estimated prevalence of more than 60% [21, 30] and an overall increasing trend by age [31], the concept of subhealth based on TCM theory has received attention in Chinese general public and health professionals. There is severe concern that public health and social problems may result if the warning signs and symptoms of subhealth are not recognized and treated properly. As a consequence of untreated subhealth, chronic disease has become the leading causes of worldwide mortality [27]. In China, chronic disease has showed a rising incidence [32, 33] and causes 85% of annual deaths [34, 35]. 55% of Chinese elderly, affected by chronic disease, has three or more concomitant persistent conditions [36].

Several previous single-centred or small-sampled studies have indicated the relationship between SDS prevalence, age, and general health status preliminarily [10, 11, 16, 17] and stated that SDS is always accompanied by other complex syndromes [15, 17, 37–40], the notions of which are partly consistent with ours. Our present large-scale study has attempted to overcome these shortcomings, allowing us to find compelling evidence of the role of spleen deficiency in the aging process and deterioration of health status.

Several limitations of our study warrant mention. This was a cross-sectional study; therefore selection bias may exist because participants were recruited from urban hospitals in Beijing and Xiamen, China. The data extracted may not be representative of the rest of China where the prevalence of SDS and distribution of symptom severity and frequency may vary from those in Beijing and Xiamen. Moreover, a cross-sectional study can only provide a basis for associations but cannot establish causality between variables such as age, health status, symptom frequency, or severity scores and prevalence of SDS.

**Figure 11:**
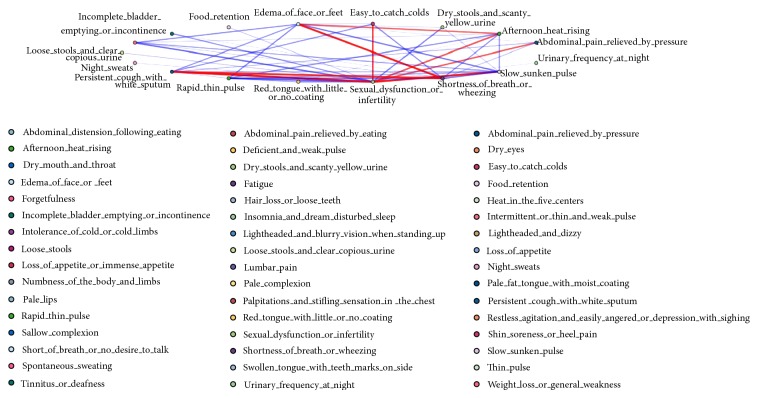
Feature selection based internal relationships of deficiency syndrome-related symptoms in SDS population. Lines of different widths represent the level of relevance between different symptoms. Several symptoms related to deficiency of qi (shortness of breath or wheezing), kidney (sexual dysfunction or infertility), lung (persistent cough with white sputum, easy to catch colds), stomach (abdominal pain relieved by pressure), yin (edema of face or feet, afternoon heat rising), and yang (slow sunken pulse) have formed tight-knit groups (lines in red) in SDS population.

Further exploration through longitudinal studies, including prospective cohort studies with long follow-up times, is needed. Results of such research can provide stronger evidence on the role of SDS in aging and health status-related processes. By testing the internal relationships of all the deficiency syndrome-related symptoms in SDS individuals based on feature selection, several symptoms have formed tight-knit groups, which indicate the complexity of evidence-based SDS model (Figure 11). In view of this, modern computational solutions, including association rules, decision tree, and complex system entropy clustering analysis algorithms, can be applied to excavate underlying core rules of SDS efficiently and objectively.

## 5. Conclusions

This study appears to support the TCM concept that spleen deficiency is an important mechanism of aging, subhealth, and chronic diseases. Early recognition of the warning signs and symptoms of SDS may lead to intervention and even prevention strategies for subhealth and chronic diseases as well as promotion of healthy aging.

## Figures and Tables

**Table 1:** Age and prevalence of SDS.

Health status	Total number	SDS, total number (%)	Age group, SDS number (%)		
			20–39 y	40–59 y	60–79 y
Healthy	366	136 (37.2)	39 (22.9)	41 (39.0)	56 (61.5)
Subhealthy	745	262 (35.2)	69 (24.0)	110 (37.4)	83 (50.6)
Chronic disease	279	160 (57.3)	21 (41.2)	43 (58.9)	96 (61.9)

**Table 2:** Health status and prevalence of SDS.

Age group, y	Total, number	SDS, total number (%)	Health status, SDS number (%)		
			Healthy	Subhealthy	Chronic disease
20–39	508	129 (25.4)	39 (22.9)	69 (24.0)	21 (41.2)
40–59	472	194 (41.1)	41 (39.0)	110 (37.4)	43 (58.9)
60–79	410	235 (57.3)	56 (61.5)	83 (50.6)	96 (61.9)

**Table 3:** SDS symptom severity scores at different ages.

Age, number	Score														
	0	1	2	3	4	5	6	7	8	9	10	11	12	13	14
20–39	162	97	81	53	32	21	23	6	6	11	9	4	2	1	0
40–59	102	65	57	52	44	49	24	30	12	9	10	11	5	2	0
60–79	48	51	47	59	51	27	23	26	12	17	13	18	16	2	0

Total	312	213	185	164	127	97	70	62	30	37	32	33	23	5	0

**Table 4:** SDS symptom frequency scores at different ages.

Age, number	Score														
	0	1	2	3	4	5	6	7	8	9	10	11	12	13	14
20–39	310	52	56	25	18	8	21	7	2	6	2	1	0	0	0
40–59	213	64	26	41	43	14	21	15	13	10	3	4	2	1	2
60–79	150	43	24	19	21	30	31	50	27	5	6	1	1	2	0

Total	673	169	106	85	82	52	73	62	42	21	11	6	3	3	2

**Table 5:** SDS symptom severity scores in varied health status.

Group, number	Score														
	0	1	2	3	4	5	6	7	8	9	10	11	12	13	14
Health	103	47	46	20	33	10	13	16	6	18	15	17	21	1	0
Subhealth	169	154	108	95	51	57	34	33	10	9	9	9	2	4	0
Chronic disease	40	12	31	49	43	30	23	13	14	10	8	7	0	0	0

Total	312	213	185	164	127	97	70	62	30	37	32	33	23	5	0

**Table 6:** SDS symptom frequency scores of SDS in varied health status.

Group, number	Score														
	0	1	2	3	4	5	6	7	8	9	10	11	12	13	14
Health	165	41	32	13	27	9	20	48	4	6	1	0	0	0	0
Subhealth	395	91	44	53	29	31	35	10	29	10	8	6	2	2	0
Chronic disease	113	37	30	19	26	12	18	4	9	5	2	0	1	1	2

Total	673	169	106	85	82	52	73	62	42	21	11	6	3	3	2

**Table 7:** Prevalence of SDS symptoms as related to health status and age.

Health status	Age group	Total (*n*)	Loose stools	Abdominal distension after meal	Sallow complexion	Loss of appetite	Weight loss and weakness
Healthy	20–39	170	58 (34.1%)	52 (30.6%)	41 (24.1%)	59 (34.7%)	37 (21.8%)
	40–59	105	36 (34.3%)	47 (44.8%)	42 (40.0%)	43 (41.0%)	53 (50.5%)
	60–79	91	52 (57.1%)	54 (59.3%)	50 (55.0%)	42 (46.2%)	52 (57.1%)

Subhealthy	20–39	287	119 (41.5%)	96 (33.4%)	67 (23.3%)	103 (36.3%)	50 (17.4%)
	40–59	294	121 (41.2%)	123 (41.8%)	159 (54.1%)	93 (31.6%)	120 (40.8%)
	60–79	164	74 (45.1%)	73 (44.5%)	92 (56.1%)	65 (39.6%)	83 (50.6%)

Chronic disease	20–39	51	29 (56.9%)	30 (58.8%)	14 (27.5%)	13 (25.5%)	37 (72.5%)
	40–59	73	30 (41.1%)	39 (53.4%)	51 (69.9%)	44 (60.3%)	47 (64.4%)
	60–79	155	78 (50.3%)	80 (51.6%)	108 (69.7%)	86 (55.5%)	80 (51.6%)
